# Are the testing needs of key European populations affected by hepatitis B and hepatitis C being addressed? A scoping review of testing studies in Europe

**DOI:** 10.3325/cmj.2016.57.442

**Published:** 2016-10

**Authors:** Jeffrey V Lazarus, Ida Sperle, Alexander Spina, Jürgen K Rockstroh

**Affiliations:** 1 CHIP, Department of Infectious Diseases, Rigshospitalet, University of Copenhagen, Copenhagen, Denmark; 2 Barcelona Institute of Global Health (ISGlobal), Hospital Clínic, University of Barcelona, Barcelona, Spain; 3 Centre for Infectious Disease Epidemiology, Austrian Agency for Health and Food Safety (AGES), Vienna, Austria; 4 Department of Medicine I, University Hospital Bonn, Bonn, Germany

## Abstract

**Aim:**

To investigate whether or not key populations affected by hepatitis B and hepatitis C are being tested sufficiently for these diseases throughout the European region.

**Methods:**

We searched MEDLINE and EMBASE for studies on HBV and HCV testing in the 53 Member States of the World Health Organization European Region following PRISMA criteria.

**Results:**

136 English-language studies from 24 countries published between January 2007 and June 2013 were found. Most studies took place in 6 countries: France, Germany, Italy, the Netherlands, Turkey, and the United Kingdom. 37 studies (27%) addressed HBV, 46 (34%) HCV, and 53 (39%) both diseases. The largest categories of study populations were people who use drugs (18%) and health care patient populations (17%). Far fewer studies focused on migrants, prison inmates, or men who have sex with men.

**Conclusions:**

The overall evidence base on HBV and HCV testing has considerable gaps in terms of the countries and populations represented and validity of testing uptake data. More research is needed throughout Europe to guide efforts to provide testing to certain key populations.

The World Health Organization (WHO) has estimated that globally 240 million people are chronically infected with the hepatitis B virus (HBV) (1), and 130 to 150 million with the hepatitis C virus (HCV) (2). According to Global Burden of Disease study findings, in 2010, hepatitis B caused almost 800 000 deaths and hepatitis C almost 500 000 deaths (3) – more than AIDS, tuberculosis, or malaria. Most of these deaths resulted from liver cirrhosis and liver cancer, both of which are common outcomes of long-term HBV and HCV infection.

Although the WHO European Region accounts for only a small proportion of the overall global burden of hepatitis B and C, both diseases are recognized as major public health threats within this region (4,5). A recent review estimated that 13.3 million adults in the WHO European Region are positive for hepatitis B surface antigen (HBsAg), a figure representing 1.8% of the adult population (6). It is estimated that adult hepatitis C RNA (HCV RNA) prevalence is 15.0 million, or 2.0% of the adult population.

The prevalence of HBV and HCV varies greatly across European countries, although gaps in the data and variations in study methodology hinder efforts to make reliable comparisons. HBsAg prevalence levels are reported to range from 0.1% (Ireland, the Netherlands) to 13.3% (Uzbekistan) (6). HCV RNA prevalence levels from 0.4% (Austria, Cyprus, Denmark, France, Germany, and the United Kingdom) to 2.9% (Romania) have been noted (7). Within the European Union, countries in the south and east appear to have lower HBV and HCV prevalence overall than countries in the northwest (8).

Among the populations thought to be heavily affected by one or more forms of viral hepatitis in Europe, the World Health Organization identifies people who inject drugs (PWID) as “the key risk group for HCV infection in most European countries,” and also calls for attention to be given to men who have sex with men (MSM) engaging in high-risk behavior (9). Migrants are another population of concern in the region (6,10,11), as are prison inmates (12,13).

The field of viral hepatitis has seen important biomedical advances in recent years. The best antiviral drugs on the market can reduce severe consequences of chronic HBV infection (14) and can cure most cases of HCV (15). While the high cost of these drugs has raised concerns about their affordability, this is not the only obstacle to treating more people. The drugs are at risk of being greatly underutilized because most people who might benefit from them remain undiagnosed (16). An analysis of data from 7 European countries concluded that only 10 to 40% of people in those countries are aware of their HCV infection (17).

There are individual and public health benefits to learning one’s hepatitis B and C status. First, people who know they have one or both of these diseases can choose to make lifestyle changes to help protect the liver, such as no longer consuming alcohol (1). It is also crucial for more people with undiagnosed HBV and HCV to learn about their condition as a prerequisite to becoming candidates for treatment. Diagnosis of HBV and HCV has important prevention implications as well. Through prevention education, people infected with both diseases can learn how to take measures to avoid onward transmission. HCV-infected people who undergo treatment and achieve a cure are no longer at risk of spreading HCV to others.

Surveying the existing published knowledge on this topic to try to gain a better understanding of testing in Europe is an important preliminary step in strengthening the public health response to the challenges of reducing the number of undiagnosed infections and engaging more people in treatment. The aim of this scoping review is to investigate whether or not key populations affected by hepatitis B and hepatitis C in Europe are being tested sufficiently for these diseases throughout the region.

## Methods

A systematic literature review was conducted on hepatitis B and C testing in the 53 Member States of the WHO European Region. The MEDLINE and EMBASE databases were searched for articles and conference abstracts published between 1 January 2007 and 30 June 2013. There was no limit set for the year of data collection. Keywords and medical subject headings (MeSH) for viral hepatitis B and C, testing and geographical scope were included in a broad search string (Supplementary material 1)(web extra material 1). The literature search was designed to identify English-language primary research articles and conference abstracts reporting on testing for hepatitis B or C in Europe. The protocol for this review was consistent with PRISMA criteria and was adapted from an earlier study that our group did on HCV among people who inject drugs (18,19).

### Study selection

**Figure 1:**
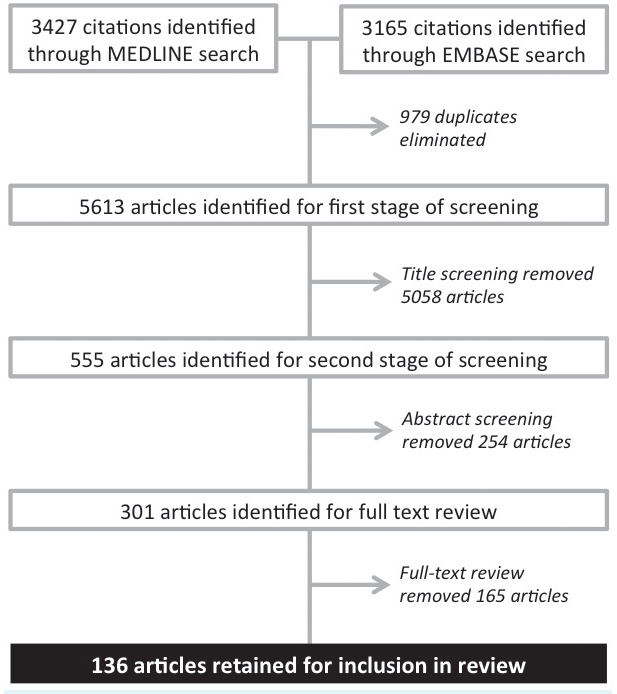
Study selection process.

The screening process for study selection is shown in Figure 1. Following the MEDLINE and EMBASE searches, duplicate results were removed and the titles and abstracts of the remaining results were screened independently by two researchers (IS and JVL) to determine whether studies presented data on hepatitis testing in Europe. The same two researchers then reviewed the full text of the 301 articles identified through this process to determine which ones met the selection criteria. In order to be included, studies needed to report on the number and proportion of study participants tested for viral hepatitis. Studies were excluded if they focused on diagnostic aspects of viral hepatitis testing, if they reported data on pooled samples only, or if they involved the testing of deceased people, organ or tissue donors, or already-diagnosed individuals. Studies that utilized multiple contemporary samples from the same person also were excluded. If two or more studies reported on the same study population, then only the most recently published study was retained.

### Data extraction

Data were extracted and inserted into an Excel spreadsheet for further analysis using basic statistical methods. These data included study country; study design; study sample size; study setting; key characteristics of the study population; number and proportion of study participants tested for viral hepatitis; type of viral hepatitis that testing was intended to detect (hepatitis B, hepatitis C, or both); viral hepatitis prevalence level or levels for those who were tested; and reported testing barriers. Decisions about which data to extract were guided by the criteria described in Box 1.

Box 1 **Data extraction criteria**

• If a study reported pre- and post-intervention data, then only the pre-intervention (baseline) data were extracted.

• If a study reported on a longitudinal cohort with data available for multiple time points, then only the baseline data were extracted.

• If a study reported on multiple sequential cross-sectional study populations, then only data for the most recent study population were extracted.

Studies were sorted into separate categories on the basis of the type of study population. If a study met the criteria for being grouped with more than one type of population, it was placed in the category most closely associated with its primary focus. If a study cohort could be disaggregated into multiple population categories, then findings for the different populations were reported separately. Studies with study populations comprised of people living with HIV as well as studies pertaining to pregnancy and assisted reproductive technology were assessed separately from other patient studies because of the large numbers of studies in these subgroups of patients.

## Results

**Figure 2:**
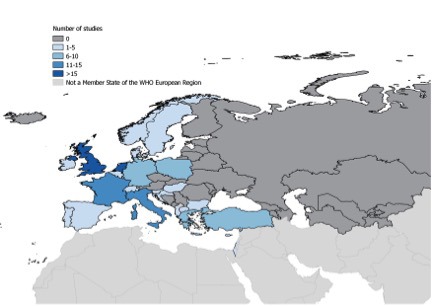
Geographical distribution of studies included in the review.

**Figure 3:**
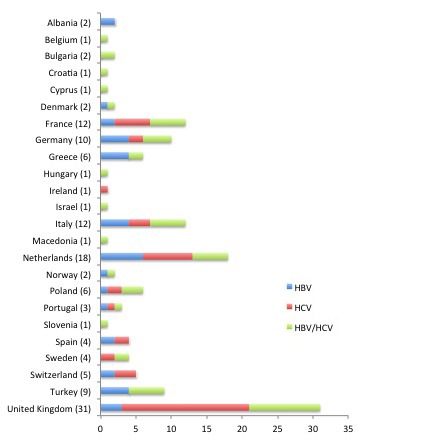
Country settings for studies included in the review.

**Figure 4:**
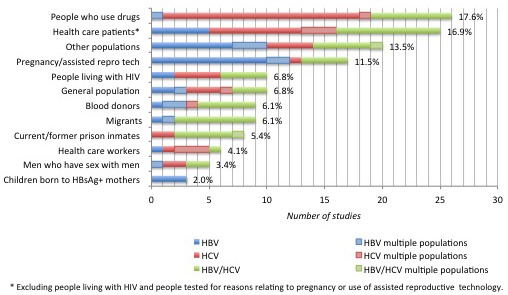
Number of studies reporting on each disease and proportion of studies by population categories in the review.

The review identified 136 studies from 24 of the WHO European Region’s 53 Member States (Figure 2, Figure 3). The countries with the largest numbers of studies were the United Kingdom (n=31), the Netherlands (n=18), France (n=12), Italy (n=12), and Germany (n=10). 37 (27.2%) studies addressed HBV, 46 (33.8%) addressed HCV, and 53 (39.0%) addressed both diseases. Studies were grouped into 12 study population categories (Figure 4). The populations most often studied were people who use drugs (17.6%), health care patient populations (16.9%), and people tested for reasons relating to pregnancy or use of assisted reproductive technology (11.5%). Although studies appeared in 78 different journals, 5 journals collectively accounted for one-quarter (27.2%) of studies: the *Journal of Viral Hepatitis* (n=15); *Epidemiology and Infection* (n=5); *the Journal of Hepatology* (n=6); *the Journal of Medical Virology* (n=5); and the *European Journal of Gastroenterology and Hepatology* (n=5). There was considerable variation in the number of studies published per year, with no obvious temporal trends.

136 studies were included in the analysis of studies grouped by study populations (20-156) (Supplementary material 2)(web extra material 2). Table 1 summarizes key findings for the 12 study populations.

**Table 1:** Key findings for study populations

**Study**		**Number of studies**	**Study sample size**		**% tested**		**Hepatitis B surface antigen (HBsAg) prevalence**		**Anti-hepatitis C virus (HCV) prevalence**	
**population**	**countries**		**range**	**median**	**range**	**median**	**range (%)**	**median (%)**	**range (%)**	**median (%)**
Blood donors (9 studies)	Albania Germany Italy Poland Turkey	Hepatitis B virus (HBV): 3 HCV: 1 HBV/ HCV: 5	801–148,320	30,716	100–100	100	0.1–9.1 (N = 8)	1.1 (N = 8)	0–0.5 (N = 6)	0.2 (N = 6)
Health care workers (6 studies)	Germany Greece Netherlands Poland	HBV: 1 HCV: 4 HBV/HCV: 1	104–9029	572	100–100	100	0.5 (N = 1)	0.5 (N = 1)	1.4–1.7 (N = 4)	1.4 (N = 4)
Health care patients (25 studies)	France Germany Greece Italy Macedonia Netherlands Poland Spain Sweden Turkey United Kingdom	HBV: 5 HCV: 11 HBV/ HCV: 9	25–90,424*	844*	13.0–100^†^	100^†^	0.1–6.2 (N = 11)	1.0 (N = 11)	0–31.5 (N = 17)	1.1 (N = 17)
People living with HIV (10 studies)	Bulgaria France Germany Netherlands Slovenia Switzerland United Kingdom	HBV: 2 HCV: 4 HBV/ HCV: 4	48–31,765	770	60.8–100	100	2.1–6.5 (N = 5)	3.9 (N = 5)	4.4–43.8 (N = 7)	10.7 (N = 7)
Migrants (9 studies)	Germany Greece Italy Netherlands United Kingdom	HBV: 2 HCV: 0 HBV/ HCV: 7	250–5000^‡^	709^‡^	0–100^§^	99.3^§^	0.6–11.7 (N = 8)^║^	3.0 (N = 8)^║^	0.4–5.6 (N = 8)^║^	1.1 (N = 8)^║^
Men who have sex with men (5 studies)	Belgium Croatia Italy Netherlands United Kingdom	HBV: 1 HCV: 2 HBV/ HCV: 2	74–5230	387	68.6–100	100	0.8–12.0 (N = 2)	6.4 (N = 2)	0.7–1.2 (N = 2)	1.0 (N = 2)
People who use drugs (26 studies)	Cyprus Denmark France Israel Italy Netherlands Sweden Switzerland United Kingdom	HBV: 1 HCV: 18 HBV/ HCV: 7	40–97,250^¶^	661^¶^	0–100**	100**	0–52.3 (N = 4)	4.8 (N = 4)	6.3–86.5 (N = 21)	50.0 (N = 21)
Current/former prison inmates (8 studies)	Bulgaria France Hungary Italy Portugal United Kingdom	HBV: 0 HCV: 2 HBV/ HCV: 6	151–318,550	550	2.6–100^††^	88.6^††^	0.7–6.7 (N = 4)	1.4 (N = 4)	4.8–77.4 (N = 7)	28.6 (N = 7)
General population (10 studies)	France Greece Italy Turkey United Kingdom	HBV: 3 HCV: 4 HBV/ HCV: 3	452–503,060	5057	100–100	100	0.5–2.5 (N = 4)	1.5 (N = 4)	1.0–5.5 (N = 4)	2.6 (N = 4)
Children born to HBsAg-positive mothers (3 studies)	Italy Netherlands	HBV: 3 HCV: 0 HBV/ HCV: 0	100–2657	2280	75.4–100	79.8	0.6–0.7 (N = 2)	0.7 (N = 2)	-	-
People tested for reasons relating to pregnancy or use of assisted reproductive technology (17 studies)	Albania Denmark Germany Greece Ireland Netherlands Norway Portugal Switzerland Turkey United Kingdom	HBV: 12 HCV: 1 HBV/ HCV: 4	206–190,141^‡‡^	3932^‡‡^	16.5–100^§§^	100^§§^	0.1–7.3 (N = 11)	0.7 (N = 11)	0.2–0.9 (N = 4)	0.4 (N = 4)
Other populations (20 studies)	Albania France Germany Greece Hungary Italy Netherlands Poland Spain Turkey United Kingdom	HBV: 10 HCV: 4 HBV/ HCV: 6	99–14,759^║║^	1000^║║^	3.7–100^¶¶^	100^¶¶^	0.1–11.9 (N = 9)***	2.1 (N = 9)***	0.1–64.3 (N = 9)***	2.8 (N = 9)***

*Based on 26 data points because one study reported separate study sample figures for hepatitis B virus (HBV) and hepatitis C virus (HCV).

†Based on 27 data points because two studies reported separate testing figures for HBV and HCV.

‡Based on 11 data points because one study reported separate study sample figures for multiple study arms.

§Based on 12 data points because (a) one study reported separate testing figures for HBV and HCV, and (b) one study reported separate testing figures for multiple study arms.

^║^Based on 9 data points because one study reported separate prevalence figures for multiple study arms.

¶Based on 27 data points because one study reported separate study sample figures for HBV and HCV.

**Based on 28 data points because (a) one study reported separate testing figures for HBV and HCV, and (b) one study reported separate testing figures for multiple study arms.

††Based on 9 data points because one study reported separate testing figures for HBV and HCV.

‡‡Based on 18 data points because one study reported separate study sample figures for HBV and HCV.

§§Based on 19 data points because two studies reported separate testing figures for HBV and HCV.

^║║^Based on 23 data points because two studies reported separate study sample figures for multiple study arms.

¶¶Based on 23 data points because two studies reported separate testing figures for multiple study arms.

***Based on 22 data points because one study reported separate prevalence figures for multiple study arms.

## Discussion

Our review of hepatitis B and C testing research identified 136 studies from 24 of the 53 countries of the WHO European Region. The study populations most frequently studied were people who use drugs, health care patient populations, and people tested for reasons relating to pregnancy or use of assisted reproductive technology.

This review found a highly uneven distribution of HBV and HCV testing-related research outputs across the countries of the WHO European Region. 6 countries accounted for more than two-thirds of the studies included in the review, and the United Kingdom alone accounted for almost one-quarter of studies. Furthermore, there appears to be a concentration of research activity in the countries of the European Union/European Free Trade Association (EU/EFTA). The only countries outside of this area with studies included in the review were Albania (2 studies), Israel (2 studies), the Former Yugoslav Republic of Macedonia (2 studies), and Turkey (9 studies). For some countries with high estimated HBV or HCV prevalence, such as Romania, Ukraine, and the Russian Federation (6), we failed to identify any studies that met review inclusion criteria.

Also, much of the research about some key populations is reported by a relatively small number of countries. For example, the 26 studies reporting on HBV/HCV testing in people who use drugs are from 9 countries, and 12 are from 1 country (the United Kingdom). While the English-language restriction that our review employed may account for some of this imbalance, the review findings nonetheless raise the question of whether there might be knowledge gaps hampering an effective response to HBV and HCV in many European countries.

Although not the main objective of the study, we found median proportions of study participants tested for HBV or HCV to be 100% across most categories of study populations. At the same time, some categories in which the median proportion tested was 100% also included studies that reported relatively low levels of testing. For example, 33.6% of study participants in a study of people who use drugs were tested for HBV (39), and 13.0% of study participants in a study of asymptomatic patients in genitourinary medicine clinics were tested for HCV (59). However, we believe that our review findings around testing uptake are of limited value in assessing testing uptake levels in these populations because few studies had the specific purpose of examining HBV and HCV testing uptake. Most instead focused on measuring HBV and HCV prevalence. In many studies with 100% testing uptake, testing was actually a requirement for study enrolment.

This review identified only 9 studies reporting on HBV and HCV testing in migrant populations, which is a matter of concern since migration is an important factor in the European hepatitis B and C epidemics. All 9 studies were from countries with large migrant populations (Germany, Greece, Italy, the Netherlands, and the United Kingdom) (157). The influx of migrants from countries with high HBV endemicity contributes considerably to the burden of chronic HBV in Europe, and chronic HBV levels of 3.7% to 6.9% have been found among migrants in 18 European countries (158). HBsAg prevalence in 8 migrant studies identified by our review ranged from 0.6% to 11.7%. Far less evidence is available regarding migration and HCV, but studies from France, the Netherlands, Spain, and the United Kingdom suggest that migrant populations may account for a sizeable proportion of HCV cases (159). A 2013 Dutch study that estimated an 0.2% national HCV antibody prevalence level indicated that the largest number of cases was in migrants from HCV-endemic countries, with fewer cases among PWID and MSM (160).

Study populations as they occur in the real world are comprised of people who belong to multiple overlapping population groups. Information about different types of demographic and behavioral factors may be required to contextualize findings relating to some specific groups. For example, one of the “people living with HIV” studies in this review found a 43.8% anti-HCV prevalence level in 48 Bulgarian study participants. While it was a heterogeneous cohort, the authors noted that all of the study participants who tested positive for anti-HCV were young men with a history of both injecting drug use and imprisonment (108).

Similarly, prison populations and PWID populations may overlap considerably. Although imprisonment puts people at a high risk of HCV infection, this is through risky behaviors that take place before or during imprisonment such as injecting drug use. While injecting drug use is likely to be the most common HCV transmission pathway among prison inmates, it is not the only one (161). This complex situation may be difficult to tease apart with existing evidence. Although our review included 8 studies enrolling current and former prison inmates, and several of those studies report high levels of injecting drug use among study participants, only 1 provides disaggregated study results for inmates who are injecting drug users and those who are not (140).

Ultimately, more data will be needed to gain insight into large-scale patterns regarding who is being tested for HBV and HCV in European countries and why. The contribution of this scoping review is to reveal a lack of evidence in the published literature for three key populations in the European HBV and HCV epidemics: migrants, prison inmates, and men who have sex with men. Although a much larger number of studies focusing on people who inject drugs were identified, the concentration of this research in a small number of countries suggests the possibility of major country-level knowledge gaps in many countries.

### Limitations

This review is subject to several limitations. Since it included only peer-reviewed studies and conference abstracts, publication bias may have significantly distorted the true picture regarding who is being tested for HBV and HCV, and in which settings. Reports from national agencies were not considered. These are likely to contribute substantially to the knowledge base regarding testing uptake. We recommend additional research to review reports from national agencies and other gray literature. Further, only articles published in English were included in this review, which may have biased our country distributions. Literature reviews of publications in other languages and of secondary literature that includes gray literature may help to provide a more complete picture. The diversity of study populations within population categories makes it challenging to interpret some of the review’s findings. Finally, there were major differences in sample sizes, which limits the comparability of findings within and across study populations.

### Conclusions

This review identified a large number of studies on HBV and HCV testing in Europe, with a wide range of populations represented in those studies as well as a highly uneven geographical distribution of studies across the countries of the WHO European Region. The overall evidence base on HBV and HCV testing in the WHO European Region appears to have considerable gaps, particularly regarding the situation in non-EU/EFTA countries and among migrant populations, prison inmates and men who have sex with men. The evidence base might be expanded considerably if key stakeholders were to coordinate studies of HBV and HCV testing behavior with national public health agencies. Finally, the issues associated with obtaining valid testing uptake data from controlled study situations suggest a need for a different approach to measuring testing uptake. Data on self-reported testing history may provide important insights.
