# Supplementary material for: Are the testing needs of key European populations affected by hepatitis B and hepatitis C being addressed? A scoping review of testing studies in Europe
Source: Croat Med J. 2016 Oct;57(5):442–56. doi: 10.3325/cmj.2016.57.442 (PMC5141462; doi:10.3325/cmj.2016.57.442)
Supplement: Supplementary material 1 [file CroatMedJ_57_s001.pdf]

Supplementary material 1: Search strings

PUBMED/MEDLINE:

(“hepatitis B” [Mesh] OR “hepatitis B” OR “HBV” OR “Hepatitis C” [Mesh] OR “hepatitis C” OR “HCV”) AND (test*[tiab] OR diagnos*[tiab] OR undiagnos*[tiab] OR “screening” [tiab] OR ”diagnostic test” [tiab] OR unidentified) NOT (haiti* OR brazil* OR iran* OR irak OR iraq* OR syria* OR malawi* OR india* OR nepal* OR japan* OR "South Africa"[MeSH Terms] OR "Developing Countries"[Mesh] OR developing countr*[tiab] OR africa* OR singapore OR australia* OR arabia*[tiab] OR aboriginal*[tiab] OR china[tiab] OR hong kong[tiab] OR Thailand OR "Africa"[Mesh] OR "Americas"[Mesh] OR "Antarctic Regions"[Mesh] OR "Arctic Regions"[Mesh] OR "Asia"[Mesh] OR "Atlantic Islands"[Mesh] OR "Australia"[Mesh] OR "Baltimore"[Mesh] OR "Boston"[Mesh] OR "Chicago"[Mesh] OR "District of Columbia"[Mesh] OR "Los Angeles"[Mesh] OR "New Orleans"[Mesh] OR "New York City"[Mesh] OR “Philadelphia"[Mesh] OR "San Francisco"[Mesh] OR "Tokyo"[Mesh] OR "Arabia"[Mesh] OR "Egypt"[Mesh] OR "Persia"[Mesh] OR "Commonwealth of Independent States"[Mesh] OR "Confederate States of America"[Mesh] OR "Korea"[Mesh] OR "Middle East"[Mesh] OR "New Guinea"[Mesh] OR "Ottoman Empire"[Mesh] OR "Russia (Pre-1917)"[Mesh] OR "Indian Ocean Islands"[Mesh] OR "Oceania"[Mesh] OR "Oceans and Seas"[Mesh] OR "Pacific Islands"[Mesh] OR "Gibraltar"[Mesh] OR "Liechtenstein"[Mesh] OR "Transcaucasia"[Mesh] OR "Vatican City"[Mesh])

EMBASE:

((Hepatitis C OR Hepatitis B OR HCV OR HBV and (test or diagnosis or undiagnosed or screening or diagnostics or diagnostic test or unidentified)) not (haiti OR brazil OR iran OR irak OR iraq OR syria OR malawi OR india OR nepal OR japan OR south africa OR developing countries OR developing country OR africa OR singapore OR australia OR arabia OR aboriginal OR china OR hong kong OR thailand OR africa or western hemishpere or antarctic regions or arctic or asia or atlantic islands or Australia or united states or japan or saudi Arabia or middle east or Egypt or iran or korea or papua new guinea or ottoman empire or Indian Ocean or pacific islands or sea or Gibraltar or Monaco)).af.

LIMITS: date: 01.01.2008-30.06.2013, humans filter and English only.
