# Supplementary material for: Are the testing needs of key European populations affected by hepatitis B and hepatitis C being addressed? A scoping review of testing studies in Europe
Source: Croat Med J. 2016 Oct;57(5):442–56. doi: 10.3325/cmj.2016.57.442 (PMC5141462; doi:10.3325/cmj.2016.57.442)
Supplement: Supplementary material 2 [file CroatMedJ_57_s002.pdf]

Supplementary material 2: studies included in review

Country	Reference	Study design	Sample size (N)	Study population	Study setting	Number of study participants tested	Disease test sought to detect	% tested	Viral hepatitis prevalence (%)	Were study partici-pants informed of test results?
	People who use drugs									
**United Kingdom**	20. Abouh-Saleh M et al. **The effectiveness of behavioral interventions in the primary prevention of hepatitis C amongst injecting drug users: A randomised controlled trial and lessons learned.** *Harm Reduction Journal* 2008;5.	Random-ised con-trolled trial	521	PWID	Drug treatment services	291	HCV	55.9	29.2 anti-HCV-positive	Yes
**United Kingdom**	21. Anderson EM et al. **Evaluation of a general practice based hepatitis C screening intervention.** Scottish Medical Journal 2009, August, 54.	Non-random-ised con-trolled trial	Interven-tion arm: 421<br>Control arm: NA	PWID	General practitioner clinics	Intervention arm: 117<br>Control arm: 0	HCV	Interven-tion arm: 27.8%<br>Control arm: 0	Intervention arm: 12.8 anti-HCV-positive; 7.7 HCV-RNA-positive<br>Control arm: none tested	Yes

Country	Reference	Study design	Sample size (N)	Study population	Study setting	Number of study participants tested	Disease test sought to detect	% tested	Viral hepatitis prevalence (%)	Were study partici-pants informed of test results?
**Sweden**	22. Blomé et al. **Minimal transmission of HCV despite persistently high transmission of hepatitis C virus in a Swedish needle exchange program.** *Journal of Viral Hepatitis*, 2011, 18: 831-839.	Retro-spective cohort study	1183	PWID	Needle exchange programme	1183	HBV, HCV	100	32.1 anti-HBc-positive<br>64.1 anti-HCV-positive	NA
**United Kingdom**	23. Brant LJ et al. **Diagnosis of acute hepatitis C virus infection and estimated incidence in low- and high-risk English populations.** *Journal of Viral Hepatitis* 2008, 15: 871-877.	Retro-spective cohort study	390	PWID	NA (serological samples obtained from laboratories)	390	HCV	100	2.1 anti-HCV-negative and HCV-RNA-positive (acute HCV infection)	NA
**United Kingdom**	24. Craine N et al. **Improving blood-borne viral diagnosis; clinical audit of the uptake of dried blood spot testing offered by a substance misuse service.** *Journal of Viral Hepatitis* 2009, 16: March.	Cross-sectional study	202	PWID	Drug treatment centre	202	HCV	100	NA	Yes

Country	Reference	Study design	Sample size (N)	Study population	Study setting	Number of study participants tested	Disease test sought to detect	% tested	Viral hepatitis prevalence (%)	Were study partici-pants informed of test results?
**United Kingdom**	25. Craine N et al. **Incidence of hepatitis C in drug injectors: the role of homelessness, opiate substitution treatment, equipment sharing and community size.** *Epidemiology and Infection* 2009, 137: 1255-1265.	Prospec-tive cohort study	700	PWID	Not specified (participants recruited from field stations including drug treatment services, needle exchange programmes and hostels for homeless people)	700	HCV	100	26.3 anti-HCV-positive	yes

Country	Reference	Study design	Sample size (N)	Study population	Study setting	Number of study participants tested	Disease test sought to detect	% tested	Viral hepatitis prevalence (%)	Were study partici-pants informed of test results?
**Italy**	26. Curcio F et al. **Epidemiological survey of hepatitis C virus infection in a cohort of patients from a ser.t in Naples, Italy.** *Journal of Addiction Medicine* 2011, 5: 43-49.	Retro-spective cohort study	1753	Patients at drug addiction centre (for injecting drug use, non-injecting drug use and alcohol consump-tion)	Drug treatment centres	1753	HCV	100	48.1 HCV-positive (marker not specified)	NA
**Cyprus**	27. Demetriou VL et al. **Hepatitis C infection among intravenous drug users attending therapy programs in Cyprus.** *Journal of Medical Virology* 2010, 82: 263-270.	Cross-sectional study	40	PWID	Detoxifica-tion centres	40	HBV, HCV	100	0 HBsAg-positive<br>50.0 anti-HCV-positive	NA

Country	Reference	Study design	Sample size (N)	Study population	Study setting	Number of study participants tested	Disease test sought to detect	% tested	Viral hepatitis prevalence (%)	Were study partici-pants informed of test results?
**France**	28. Foucher J et al. **FibroScan used in street-based outreach for drug users is useful for hepatitis C virus screening and management: a prospective study.** *Journal of Viral Hepatitis*, 2009, 16: 121-131.	Prospec-tive cohort study	198	People who use drugs (injecting and non-injecting)	Not specified (participants recruited through street-based outreach)	154	HCV	77.8	9.8 HCV-RNA-positive	Yes
**United Kingdom**	29. Hope V et al. **Frequency, factors and costs associated with injection site infections: Findings from a national multi-site survey of injecting drug users in England.** *BMC Infectious Diseases* 2008, 8:120.	Cross-sectional study	1058	PWID	Services for drug users and community settings	1058	HBV, HCV	100	32.0 anti-HBc-positive<br>53.0 anti-HCV-positive	NA
**United Kingdom**	30. Hope V et al. **Hepatitis C infection among recent initiates to injecting in England 2000-2008: Is a national HCV action plan making a difference?** *Journal of Viral Hepatitis* 2012, 19: 55-64.	Cross-sectional study	3463	PWID	Services for drug users	3463	HCV	100	17.9 anti-HCV-positive	Yes

Country	Reference	Study design	Sample size (N)	Study population	Study setting	Number of study participants tested	Disease test sought to detect	% tested	Viral hepatitis prevalence (%)	Were study partici-pants informed of test results?
**United Kingdom**	31. Hope VD et al. **Measuring the incidence, prevalence and genetic relatedness of hepatitis C infections among a community recruited sample of injecting drug users, using dried blood spots.** *Journal of Viral Hepatitis* 2011,18: 262-270.	Cross-sectional study	299	PWID	None (participants recruited through respondent-driven sampling)	299	HCV	100	59.2 anti-HCV-positive	NA
**France**	32. Jauffret-Roustide M et al. **A national cross-sectional study among drug-users in France: Epidemiology of HCV and highlight on practical and statistical aspects of the design.** *BMC Infectious Diseases* 2009, 9: 113.	Cross-sectional study	1462	People who use drugs (injecting and non-injecting)	Drug user services and general practitioner practices	1155[1]	HCV	79[2]	59.8 anti-HCV-positive	Yes

[1] Approximation.
[2] Approximation.

Country	Reference	Study design	Sample size (N)	Study population	Study setting	Number of study participants tested	Disease test sought to detect	% tested	Viral hepatitis prevalence (%)	Were study partici-pants informed of test results?
**Sweden**	33. Lidman C et al. **Hepatitis C infection among injection drug users in Stockholm Sweden: prevalence and gender.** *Scandinavian Journal of Infectious Diseases* 2009, 41: 679-684.	Cross-sectional study	310	PWID	Drug user services including inpatient and outpatient services	310	HBV, HCV	100	52.3 HBsAg-positive<br>86.5 anti-HCV-positive	NA
**Nether-lands**	34. Lindenburg CEA et al. **Hepatitis C testing and treatment among active drug users in Amsterdam: Results from the DUTCH-C project.** *European Journal of Gastroenterology and Hepatology* 2011, 23: January.	Prospec-tive cohort study	497	People who use drugs (injecting and non-injecting)	Public Health Service Amsterdam	449	HCV	90.3	59.5 anti-HCV-positive<br>29.8 HCV-RNA-positive	Yes
**Israel**	35. Loebstein R et al. **Hepatitis C, B, and human immunodeficiency virus infections in illicit drug users in Isreal: Prevalence and risk factors.** *Israel Medical Association Journal* 2008, 10: November.	Cross-sectional study	1443	People who use drugs (injecting and non-injecting)	National Center for Diagnosis of Drug Addicts	1443	HBV, HCV	100	3.5 HBsAg-positive<br>35.7 anti-HCV-positive	Yes

Country	Reference	Study design	Sample size (N)	Study population	Study setting	Number of study participants tested	Disease test sought to detect	% tested	Viral hepatitis prevalence (%)	Were study partici-pants informed of test results?
**United Kingdom**	36. McDonald SA et al. **Examination of the risk of reinfection with hepatitis C among injecting drug users who have been tested in Glasgow.** *International Journal of Drug Policy* 2012, 23: 353-357.	Cross-sectional study	97,250	PWID	NA (data obtained from Health Protection Scotland database of hepatitis C testing activity)	97,250	HCV	100	NA	NA
**Denmark**	37. Mössner BK et al. **Decline in hepatitis B infection observed after 11 years of regional vaccination among danish drug users.** *Journal of Medical Virology* 2010, 82:1635–1639.	Cross-sectional study	374	PWID	Drug treatment centres	374	HBV, HCV	100	50.2 anti-HBc-positive<br>66.8 anti-HCV-positive	NA

Country	Reference	Study design	Sample size (N)	Study population	Study setting	Number of study participants tested	Disease test sought to detect	% tested	Viral hepatitis prevalence (%)	Were study partici-pants informed of test results?
**United Kingdom**	38. O'Leary MC et al. **The association between alcohol use and hepatitis C status among injecting drug users in Glasgow.** *Drug and Alcohol Dependence* 2012, 123: 180-189.	Cross-sectional study	808	PWID	Pharmacies; fixed-site needle exchange programmes	780	HCV	96.5	64.9 anti-HCV-positive	Participants did not get their results; those who wished to know their HCV status were referred to the appropriate services
**Nether-lands**	39. Schreuder I et al. **Seroprevalence of HIV, hepatitis B and hepatitis C among opioid drug users on methadone treatment in the netherlands.** *Harm Reduction Journal* 2010, 7.	Cross-sectional study	HBV: 2024<br>HCV: 2566	People who use drugs (injecting and non-injecting)	Methadone clinics	HBV: 680<br>HCV: 1359	HBV, HCV	HBV: 33.6<br>HCV: 53.0	33.1 anti-HBc-positive<br>25.8 anti-HCV-positive	Yes

Country	Reference	Study design	Sample size (N)	Study population	Study setting	Number of study participants tested	Disease test sought to detect	% tested	Viral hepatitis prevalence (%)	Were study partici-pants informed of test results?
**Switzer-land**	40. Senn O et al. **Determinants of successful chronic hepatitis C case finding among patients receiving opioid maintenance treatment in a primary care setting.** *Addiction* 2009, 104: 2033-2038.	Retro-spective cohort study	387	PWID	General practitioner practices	327	HCV	84.5	41.6 anti-HCV-positive	NA
**United Kingdom**	41. Stephens BP et al. **Is it Worth Testing Unstable Drug Users For Hepatitis C?** Gut Conference: British Association for the Study of the Liver Annual Meeting, BASL 2011 London United Kingdom. Conference Publication: (var pagings) 2011; 60(pp A31-A32): September	Cross-sectional study	661	PWID	Drug treatment services	661	HCV	100	28.1 anti-HCV-positive	Yes (if patient returned for result)

Country	Reference	Study design	Sample size (N)	Study population	Study setting	Number of study participants tested	Disease test sought to detect	% tested	Viral hepatitis prevalence (%)	Were study partici-pants informed of test results?
**United Kingdom**	42. Tait JM et al. **Dry blood spot testing for hepatitis C in people who injected drugs: Reaching the populations other tests cannot reach.** *Journal of Hepatology* 2013, 58: S63-S227.	Prospec-tive cohort study	946	PWID	Drug treatment services	946	HCV	100	31.2 anti-HCV-positive	Yes (if patient returned for result)
**Nether-lands**	43. Van den Berg CH et al. **Never injected, but hepatitis C virus-infected: a study among self-declared never-injecting drug users from the Amsterdam Cohort Studies.** *Journal of Viral Hepatitis* 2009, 16: 568-577.	Retro-spective cohort study	364	People who use non-injecting drugs	Public Health Service Amsterdam	352	HCV	96.7	6.3 anti-HCV-positive	NA

Country	Reference	Study design	Sample size (N)	Study population	Study setting	Number of study participants tested	Disease test sought to detect	% tested	Viral hepatitis prevalence (%)	Were study partici-pants informed of test results?
**Nether-lands**	44. van Houdt R et al. **Unexpectedly high proportion of drug users and men having sex with men who develop chronic hepatitis B infection.** *Journal of Hepatology* 2012, 57:529-533.	Retro-spective cohort study	1268	People who use drugs	Public Health Service Amsterdam	1268	HBV	100	6.0 HBsAg-positive<br>52.9 anti-HBc-positive<br>64.0 anti-HCV-positive	No
**Switzer-land**	45. Witteck A et al. **Management of hepatitis C virus (HCV) infection in drug substitution programs.** *Swiss Medical Weekly* 2011, 141: w13193.	Cross-sectional study	398	People who use drugs (injecting and non-injecting)	Drug substitution programmes	394	HCV	99.0	76.7 anti-HCV-positive<br>44.4 HCV-RNA-positive	Yes

Country	Reference	Study design	Sample size (N)	Study population	Study setting	Number of study participants tested	Disease test sought to detect	% tested	Viral hepatitis prevalence (%)	Were study partici-pants informed of test results?
	***Health care patients*[3]**									
**France**	46. Ansemant T et al. **Usefulness of routine hepatitis C and hepatitis B serology in the diagnosis of recent-onset arthritis. Systmatic prospective screening in all patients seen by the rheumatologists of a defined area - Brief report.** *Joint Bone Spine,* 2012, 2012: 268-270.	Cross-sectional study	233	Patients with recent-onset arthritis	Rheumatol-ogy practices	233	HCV	100	0.4 anti-HCV-positive	Yes

[3] Excluding people living with HIV and people tested for reasons relating to pregnancy or use of assisted reproductive technology.

Country	Reference	Study design	Sample size (N)	Study population	Study setting	Number of study participants tested	Disease test sought to detect	% tested	Viral hepatitis prevalence (%)	Were study partici-pants informed of test results?
**United Kingdom**	47. Arnold DT et al. **Should patients with abnormal liver function tests in primary care be tested for chronic viral hepatitis: cost minimisation analysis based on a comprehensively tested cohort.** *BMC Family Practice* 2011, 12:9.	Prospec-tive cohort study	1236	Patients with abnormal liver function test results	Primary care clinics	1236	HBV, HCV	100	0.7 HBsAg-positive<br>0.3 anti-HCV-positive	NA
**Spain**	48. Barril G et al. **Occult hepatitis C virus infection among hemodialysis patients.** *Journal of the American Society of Nephrology* 2008, 19: 2288-2292.	Cross-sectional study	109	Haemodialy-sis patients with abnormal liver function test results	Haemodialy-sis unit	109	HCV	100	45.0 HCV-RNA-positive in peripheral blood mononuclear cells<br>(all patients were anti-HCV-negative and serum HCV-RNA-negative)	NA

Country	Reference	Study design	Sample size (N)	Study population	Study setting	Number of study participants tested	Disease test sought to detect	% tested	Viral hepatitis prevalence (%)	Were study partici-pants informed of test results?
**Macedo-nia**	49. Bosevska G et al. **Screening for hepatitis B, C and HIV infection among patients on haemodialysis (cross sectional analysis among patients from two dialysis units in the period January to July 2005).** *Prilozi* 2009, 30: 159-174.	Cross-sectional study	178	Haemodialy-sis patients with end-stage renal disease	Haemodialy-sis units	178	HBV, HCV	100	1.7 HBsAg-positive<br>3.9 anti-HBs-positive<br>7.3 anti-HBc-positive<br>31.5 anti-HCV-positive<br>21.4 HCV-RNA-positive	Yes

Country	Reference	Study design	Sample size (N)	Study population	Study setting	Number of study participants tested	Disease test sought to detect	% tested	Viral hepatitis prevalence (%)	Were study partici-pants informed of test results?
**Spain**	50. Cano J et al. **Screening for Hepatitis B Virus in a Department of Clinical Oncology in Spain.** European Journal of Cancer Conference: 2011 European Multidisciplinary Cancer Congress Stockholm Sweden Conference Start: 0110923 onference End: 20110927 Conference Publication: (var pagings) 2011; 47 (pp S250): September	Cross-sectional study	128	Patients with solid tumours	Hospital oncology department	128	HBV	100	3.9 anti-HBc-positive	NA
**France**	51. Chevaux JB et al. **Prevalence of hepatitis B and C and risk factors for nonvaccination in inflammatory bowel disease patients in Northeastern France.** *Inflammatory Bowel Disease*, 2010, 16.	Prospec-tive cohort study	315	Patients with inflamma-tory bowel disease	Hospital hepato-gastroenter-ology department	315	HBV, HCV	100	1.0 HBsAg-positive<br>2.5 anti-HBc-positive<br>1.0 anti-HCV-positive	NA

Country	Reference	Study design	Sample size (N)	Study population	Study setting	Number of study participants tested	Disease test sought to detect	% tested	Viral hepatitis prevalence (%)	Were study partici-pants informed of test results?
**Poland**	52. Flisiak R et al. **Prevalence and risk factors of HCV infection in Poland.** *European Journal of Gastroenterology and Hepatology* 2011, 23: 1213-1217.	Cross-sectional study	8901	Patients in various inpatient and outpatient health care settings	Hospitals and outpatient clinics	8901	HCV	100	1.9 anti-HCV-positive<br>0.8 HCV-RNA-positive	NA
**Poland**	53. Ganczak M et al. **Infections with HBV, HCV and HIV in patients admitted to the neurosurgical department of a teaching hospital.** *Neurologia i Neurochirurgia Polska* 2008, 42:231-237.	Cross-sectional study	100	Neurosur-gical patients	Hospital neurosur-gical ward	100	HBV, HCV	100	2.0 HBsAg-positive<br>2.0 anti-HCV-positive	No<br>(testing was anony-mous)
**Poland**	54. Ganczak M et al. **Seroprevalence of hepatitis C virus infection among surgical nurses, their patients and blood donation candidates in Poland.** *Journal of Hospital Infection* 2012, 82: 266-270.	Cross-sectional study	1118	Female patients in surgical and gynaeco-logical wards	Hospital surgical and gynaeco-logical wards	1118	HCV	100	1.1 anti-HCV-positive	Yes

Country	Reference	Study design	Sample size (N)	Study population	Study setting	Number of study participants tested	Disease test sought to detect	% tested	Viral hepatitis prevalence (%)	Were study partici-pants informed of test results?
**France**	55. Guennoc X et al. **Is screening for hepatitis B and hepatitis C useful in patients with recent-onset polyarthritis? The ESPOIR cohort study.** *The Journal of Rheumatology* 2009; 36 (7): 1407-1413	Prospec-tive cohort study	813	Patients with recent-onset polyarthritis	Hospitals	808	HBV, HCV	99.3	0.1 HBsAg-positive<br>0.9 anti-HCV-positive	NA
**Italy**	56. Ippolito AM et al. **Unawareness of HBV infection among inpatients in a Southern Italian hospital.** *Journal of Viral Hepatitis* 2011, 18:e206-e211.	Cross-sectional study	25	Hospital inpatients	Hospital inpatient wards	25	HBV	100	1.2 HBsAg-positive	Yes
**Greece**	57. Kakisi OK et al. **Prevalence of hepatitis B, hepatitis C, and HIV infections among patients in a psychiatric hospital in Greece.** *Psychiatric Services* 2009, 60: 1269-1272.	Retro-spective cohort study	805	Psychiatric hospital inpatients	Psychiatric hospital inpatient ward	HBV: 803<br>HCV: 793	HBV, HCV	HBV: 99.8<br>HCV: 98.5	2.0 HBsAg-positive<br>9.0 anti-HCV-positive	NA

Country	Reference	Study design	Sample size (N)	Study population	Study setting	Number of study participants tested	Disease test sought to detect	% tested	Viral hepatitis prevalence (%)	Were study partici-pants informed of test results?
**Spain**	58. Marcos M et al. **Chronic hepatitis B virus infection in Sjögren's syndrome.** Prevalence and clinical significance in 603 patients. *Autoimmunity Reviews* 2009, 8: 616-620.	Cross-sectional study	603	Patients with Sjogren syndrome	Hospital autoimmune diseases department	603	HBV	100	0.8 HBsAg-positive	NA
**United Kingdom**	59. McClean H et al. **National audit of asymptomatic screening in UK genitourinary medicine clinics: case-notes audit.** *International Journal of STD and AIDS* 2010, 21: 506-511.	Cross-sectional study	HBV: 4297<br>HCV: 4420	Patients seen in genitouri-nary medicine clinics	Genitouri-nary medicine clinics	HBV: 1122<br>HCV: 566	HBV, HCV	HBV: 25.7<br>HCV: 13.0	NA	NA

Country	Reference	Study design	Sample size (N)	Study population	Study setting	Number of study participants tested	Disease test sought to detect	% tested	Viral hepatitis prevalence (%)	Were study partici-pants informed of test results?
**Germany**	60. Muche M et al. **Prevalence and risk factors for hepatitis B and C among patients attending a german emergency department - Is a risk assessment questionnaire a useful pre-screening tool?** *Journal of Hepatology Conference*: 48th Annual Meeting of the European Association for the Study of the Liver, International Liver Congress 2013 Amsterdam.	Cross-sectional study	1942	Hospital emergency department patients	Hospital emergency department	1942	HBV, HCV	100	0.5 HbsAg-positive<br>9.9 anti-HBc-positive<br>1.6 anti-HCV-positive	NA
**Turkey**	61. Okan V et al. **Prevalence of hepatitis B and C viruses in patients with lymphoproliferative disorders.** *International Journal of Hematology* 2008, 88: 403-408.	Cross-sectional study	1014	Patients with lympho-proliferative disorders	Haemotol-ogy clinic	334	HBV, HCV	100	6.2 HBsAg-positive<br>2.7 anti-HCV-positive	NA

Country	Reference	Study design	Sample size (N)	Study population	Study setting	Number of study participants tested	Disease test sought to detect	% tested	Viral hepatitis prevalence (%)	Were study partici-pants informed of test results?
**Turkey**	62. Ozaslan E et al. **Occult hepatitis B virus infection in Turkish healthy individuals.** *European Journal of Gastroenterology and Hepatology* 2009, 21:1436-1440.	Prospec-tive cohort study	347	Patients presenting for nonspecific complaints or routine medical check-ups	Not specified	347	HBV	100	NA	NA
**Italy**	63. Palazzi C. **Hepatitis C virus infection in Italian patients with fibromyalgia.** *Clinical Rheumatology* 2008, 27: 101-103.	Cross-sectional study	152	Patients with fibromyalgia	Rheumatol-ogy outpatient clinic	152	HCV	100	4.6 anti-HCV-positive	NA
**Germany**	64. Ross RS et al. **Transmission of hepatitis C virus in an orthopedic hospital ward.** *Journal of Medical Virology* 2009, 81: 249-257.	Retro-spective cohort study	135	Patients in a hospital orthopaedic ward	Hospital orthopaedic ward	122	HCV	90.4	4.4 HCV-RNA-positive	Yes

Country	Reference	Study design	Sample size (N)	Study population	Study setting	Number of study participants tested	Disease test sought to detect	% tested	Viral hepatitis prevalence (%)	Were study partici-pants informed of test results?
**Sweden**	65. Sjöberg K et al. **Prevalence of hepatitis C in Swedish diabetics is low and comparable to that in health care workers.** *European Journal of Gastroenterology and Hepatology* 2008, 20:135-138.	Cross-sectional study	874	Patients with diabetes	Hospital endocrinol-ogy department	874	HCV	100	0.7 anti-HCV-positive	NA
**Nether-lands**	66. Slavenburg S et al. **Prevalence of hepatitis C in the general population in the Netherlands.** *The Netherlands Journal of Medicine* 2008, 66: 13-17.	Cross-sectional study	2200	People who visited general practices in urban eastern Netherlands	General practices	2200	HCV	100	0.7 anti-HCV-positive	No
**France**	67. Spenatto N et al. **Hepatitis B screening: Who to target? A French sexually transmitted infection clinic experience.** *Journal of Hepatology* 2013, 58: 690-697.	Cross-sectional study	7692	Asympto-matic people seeking STI screening	STI clinic	6194	HBV	80.5	0.8 HBsAg-positive	Yes

Country	Reference	Study design	Sample size (N)	Study population	Study setting	Number of study participants tested	Disease test sought to detect	% tested	Viral hepatitis prevalence (%)	Were study partici-pants informed of test results?
**United Kingdom**	68. Tweed E et al. **Hepatitis C testing in sexual health services in England, 2002e7: results from sentinel surveillance.** *Sexually Transmitted Infections* 2010, 86: 126-130.	Retro-spective cohort study	90,424	Patients seeking sexual health services	Sexual health clinic	90,424	HCV	100	3.2 anti-HCV-positive	NA
**Germany**	69. Vermehren J et al. **High prevalence of anti-HCV antibodies in two metropolitan emergency departments in Germany: a prospective screening analysis of 28,809 patients.** *PLoS One* 2012, 7: e41206.	Retro-spective cohort study	28,809	Hospital emergency department patients	Hospital emergency departments	28,809	HCV	100	2.6 anti-HCV-positive	Yes
**Sweden**	70. Ydreborg M et al. **Look-back screening for the identification of transfusion-induced hepatitis C virus infection in Sweden.** *Scandinavian Journal of Infectious Diseases* 2011, 43: 522-527.	Cross-sectional study	13,573	People who received blood transfusions prior to 1992	None (serological samples obtained from laboratories)	13,573	HCV	100	0.9 anti-HCV-positive	NA

Country	Reference	Study design	Sample size (N)	Study population	Study setting	Number of study participants tested	Disease test sought to detect	% tested	Viral hepatitis prevalence (%)	Were study partici-pants informed of test results?
	Other populations									
**United Kingdom**	71. Balogun MA et al. **Hepatitis B virus transmission in pre-adolescent schoolchildren in four multi-ethnic areas of England.** *Epidemiology & Infection*2013, 141: 916-925.	Cross-sectional study	5661	Children aged 7-11	NA	5334	HBV	94.2	0.26 anti-HBc-positive	NA

Country	Reference	Study design	Sample size (N)	Study population	Study setting	Number of study participants tested	Disease test sought to detect	% tested	Viral hepatitis prevalence (%)	Were study partici-pants informed of test results?
**United Kingdom**	72. Brown AE et al. **Prevalence of markers for HIV, hepatitis B and hepatitis C infection in UK military recruits.** *Epidemiology and Infection* 2011, 139: August.	Cross-sectional study	14,759	Military recruits	NA	14,663	HBV, HCV	99.3	0.4 HBsAg-positive<br>3.6 anti-HBc-positive<br>0.06 anti-HCV-positive	No<br>(testing was anonymous)
**Germany**	73. Cai W et al. **Hepatitis B infections among children and adolescents in Germany.** *The Pediatric Infectious Disease Journal* 2011, 30.	Cross-sectional study	14,747[4]	Children aged 3–17	NA	13,065	HBV	88.6	0.5 anti-HBc-positive	NA

[4] Approximation.

Country	Reference	Study design	Sample size (N)	Study population	Study setting	Number of study participants tested	Disease test sought to detect	% tested	Viral hepatitis prevalence (%)	Were study partici-pants informed of test results?
**Spain**	74. Castillo I et al. **Hepatitis C virus infection in the family setting of patients with occult hepatitis C** *Journal of Medical Virology* 2009, 81: 1198-1203.	Cross-sectional study	218	Family members of people with occult HCV infection or chronic HCV infection	NA	218	HCV	100	6.4 anti-HCV-positive<br>4.1 HCV-RNA-positive	NA
**United Kingdom**	75. Coyne KM et al. **Sexual health of adults working in pornographic films.** *International Journal of STD and AIDS* 2009, 20: 508-509.	Cross-sectional study	115	Adult film industry actors	Sexual health clinic	115	HBV, HCV	100	0 HBV-positive (marker not specified)<br>0 HCV-positive (marker not specified)	NA

Country	Reference	Study design	Sample size (N)	Study population	Study setting	Number of study participants tested	Disease test sought to detect	% tested	Viral hepatitis prevalence (%)	Were study partici-pants informed of test results?
**Nether-lands**	76. Cuypers WJ et al. **High prevalence of HIV, other sexually transmitted infections and risk profile in male commercial sex workers who have sex with men in the Netherlands.** Sexually Transmitted Infections Conference: 19th Biennial Conference of the International Society for Sexually Transmitted Diseases Research Quebec City, QC Canada 2011. Conference Publication: /var pagings 011; 87 (pp A127): July.	Retro-spective cross-sectional study	99	Male commercial sex workers who have sex with men	Not specified	99	HBV	100	8.1 HBsAg-positive	NA

Country	Reference	Study design	Sample size (N)	Study population	Study setting	Number of study participants tested	Disease test sought to detect	% tested	Viral hepatitis prevalence (%)	Were study partici-pants informed of test results?
**United Kingdom**	77. Hickman M et al. **Increasing the uptake of hepatitis C virus testing among injecting drug users in specialist drug treatment and prison settings by using dried blood spots for diagnostic testing: a cluster randomized controlled trial.** Journal of Viral Hepatitis 2008, 15: 250-254.	Cluster-random-ised con-trolled trial	Interven-tion arm: 6550<br>Control arm: 5800	Drug users and male prison inmates (not disaggre-gated)	Drug treatment services and prisons	Intervention arm: 1174<br>Control arm: 535	HCV	Interven-tion arm: 17.9%<br>Control arm: 9.2%	31.0 anti-HCV-positive (intervention and control patients combined)	NA
**Turkey**	78. Karaosmanoglu HK et al. **Seroprevalence of hepatitis B: do blood donors represent the general population?** *Journal of Infection in Developing Countries* 2012, 6:181-183.	Retro-spective cohort study	10,903 (9,949 blood donors, 954 pre-marital screening partici-pants)	Healthy individuals undergoing premarital screening	NA	954	HBV	100	3.4 HBsAg-positive	NA

Country	Reference	Study design	Sample size (N)	Study population	Study setting	Number of study participants tested	Disease test sought to detect	% tested	Viral hepatitis prevalence (%)	Were study partici-pants informed of test results?
**Albania**	79. Katsanos KH et al. **Hepatitis B remains a major health priority in Western Balkans: Results of a 4-year prospective Greek–Albanian collaborative study.** *European Journal of Internal Medicine* 2009, 20: 698-702.	Prospec-tive cohort study	410	Albanians aged 14–20	Not specified	410	HBV	100	11.9 HBsAg-positive	NA
**France**	80. Meffre C. **Prevalence of hepatitis B and hepatitis C virus infections in France in 2004: Social factors are important predictors after adjusting for known risk factors.** *Journal of Medical Virology* 2010, 82: 546-555.	Cross-sectional study	14,416	French metropoli-tan residents aged 18–80	NA	14,416	HBV, HCV	100	7.3 anti-HBc-positive<br>0.8 anti-HCV-positive	NA
**Poland**	81. Pazdiora P et al. **Screening family and sexual contacts of HBsAg+ persons in the Pilsen region.** *Epidemiology, Mikrobiology, Immunology* 2012, 61: 51-57.	Cross-sectional study	1017	Family and sexual contacts of HBsAg-positive persons	NA	908	HBV	89.3	18.0 HBC-positive (marker not specified)	NA

Country	Reference	Study design	Sample size (N)	Study population	Study setting	Number of study participants tested	Disease test sought to detect	% tested	Viral hepatitis prevalence (%)	Were study partici-pants informed of test results?
**Albania**	82. Resuli B et al. **Epidemiology of hepatitis B virus infection in Albania.** *World Journal of Gastroenterology* 2009, 15:849-852.	Cross-sectional study	1576	Heteroge-neous study sample included high school students, university students and military personnel	High schools; universities; military facilities; blood donation centre	1576	HBV	100	10.2 HBsAg-positive<br>29.1 anti-HBs-positive	NA

Country	Reference	Study design	Sample size (N)	Study population	Study setting	Number of study participants tested	Disease test sought to detect	% tested	Viral hepatitis prevalence (%)	Were study partici-pants informed of test results?
**France**	83. Sahajian F et al. **A randomized trial of viral hepatitis prevention among underprivileged people in the Lyon area of France.** Journal of Public Health 2011, 33: 182-192.	Random-ised con-trolled trial	Interven-tion 1: 222<br>Interven-tion 2: 243<br>Control: 811	Residents of homeless shelters	Homeless shelters	Intervention 1: 161<br>Intervention 2: 188<br>Control: 30	HBV, HCV	Interven-tion 1: 72.5<br>Interven-tion 2: 77.4<br>Control: 3.7	Intervention 1: 2.1 HBsAg-positive; 3.2 anti-HCV-positive<br>Intervention 2: 4.8 HBsAg-positive, 2.8 anti-HCV-positive<br>Control: 0 HBsAg-positive, 0 anti-HCV-positive	NA

Country	Reference	Study design	Sample size (N)	Study population	Study setting	Number of study participants tested	Disease test sought to detect	% tested	Viral hepatitis prevalence (%)	Were study partici-pants informed of test results?
**Greece**	84. Stamouli M et al. **Prevalence of Hepatitis B among Greek Naval Recruits.** Clinical Chemistry and Laboratory Medicine Conference: Congress of the Swiss Union of Laboratory Medicine, SULM - Swiss MedLab 2012 Berne Switzerland Conference Publication: (var pagings) 2012; 50 (5): May.	Cross-sectional study	1000	Military recruits	NA	1000	HBV	100	0.1 HBsAg-positive<br>74.5 anti-HBs-positive<br>0.9 anti-HBc-positive	NA
**United Kingdom**	85. Taylor A et al. **Prevalence and determinants of hepatitis C virus infection among female drug injecting sex workers in Glasgow.** *Harm Reduction Journal* 2008, 5.	Cross-sectional study	114	Street-based sex workers	Health and social services drop-in centre	103	HCV	90.4	64.3 anti-HCV-positive	No<br>(testing was anonymous)

Country	Reference	Study design	Sample size (N)	Study population	Study setting	Number of study participants tested	Disease test sought to detect	% tested	Viral hepatitis prevalence (%)	Were study partici-pants informed of test results?
**Hungary**	86. Treso B et al. **Prevalence and Correlates of HCV, HVB, and HIV infection among prison inmates and staff, Hungary.** *Journal of Urban Health* 2012, 89: 108-116.	Cross-sectional study	1066	Prison staff	Prisons	1066	HBV, HCV	100	0.4 HBsAg-positive<br>0.5 anti-HCV-positive	NA
**Italy**	87. Trevisan A et al. **Prevalence of Markers for Hepatitis B Virus and Vaccination Compliance Among Medical School Students in Italy.** *Infection control and hospital epidemiology* 2008, 29 : 1189-1191.	Cross-sectional study	2361	Students attending graduate courses at a university medical school	University medical school	2361	HBV	100	NA	Yes
**Nether-lands**	88. Urbanus AT et al. **People with multiple tattoos and/or piercings are not at increased risk for HBV or HCV in the Netherlands.** *Plos One* 2011, 6 : e24736.	Cross-sectional study	434	Tattoo artists, piercers, and people with multiple tattoos and/or piercings	tattoo conventions; tattoo and/or piercing shops; STI outpatient clinic	434	HBV, HCV	100	4.2 anti-HBc-positive<br>0.2 anti-HCV-positive	NA

Country	Reference	Study design	Sample size (N)	Study population	Study setting	Number of study participants tested	Disease test sought to detect	% tested	Viral hepatitis prevalence (%)	Were study partici-pants informed of test results?
**Germany**	89. Walz A et al. **Vertical transmission of HBV virus (HBV) from mothers negative for HBV surface antigen and positive for antibody to HBV core antigen.** *The Journal of Infectious Diseases* 2009, 200:1227-1231.	Cross-sectional study	105	Children of women who are HBsAg-positive and anti-HBc-positive	Maternity clinics	105	HBV	100	1.9 HBsAg-positive<br>3.8 anti-HBc-positive<br>4.8 HBV-DNA-positive	Yes
**Nether-lands**	90. Zuure FR et al. **Using mass media and the internet as tools to diagnose hepatitis C infections in the general population.** *American Journal of Preventive Medicine* 2011 40: 345-352.	Cross-sectional study	1480	Self-identified as belonging to one of several groups at risk for HCV (e.g. blood transfusion prior to 1992, HCV-endemic country of birth)	NA	420	HCV	28.4	3.6 anti-HCV-positive	Yes, but informed online so would need to check themselves

Country	Reference	Study design	Sample size (N)	Study population	Study setting	Number of study participants tested	Disease test sought to detect	% tested	Viral hepatitis prevalence (%)	Were study partici-pants informed of test results?
People tested for reasons relating to pregnancy or use of assisted reproductive technology										
**Germany**	91. Bissinger A.L et al. **Frequency of hepatitis B antigen positive pregnant women with high viral loads detected during routine antenatal care in Germany.** *Journal of Hepatology* 2013, 58:S229-S407.	Cross-sectional study	41,942	Pregnant women	Hospitals	41,942	HBV	100	0.6 HBsAg-positive	NA
**Norway**	92. Bjerke S et al. **Infectious immune status in an obstetric population of Pakistani immigrants in Norway.** *Scandinavian Journal of Public Health* 2011, 39:464.	Cross-sectional study	206	Pregnant women (immigrant)	Hospitals	206	HBV	100	11.0 anti-HBc-positive	Yes
**Switzer-land**	93. Frischknecht F et al. **Serological testing for infectious diseases in pregnant women: are the guidelines followed?** *Swiss Medical Weekly* 2011, 140: 13138.	Cross-sectional study	723	Pregnant women	Hospital labour ward	703	HBV	97.2	0.7 anti-HBs-positive	NA

Country	Reference	Study design	Sample size (N)	Study population	Study setting	Number of study participants tested	Disease test sought to detect	% tested	Viral hepatitis prevalence (%)	Were study partici-pants informed of test results?
**Turkey**	94. Gun I et al. **Seroprevalence among Turkish pregnant women.** *HealthMED Journal* 2012, 6: 2012.	Retro-spective cohort study	1537	Pregnant women	Outpatient clinic	1537	HBV, HCV	100	2.3 anti-HBs-positive<br>0.2 anti-HCV-positive	NA
**Denmark**	95. Harder KM et al. **Universal screening for hepatitis B among pregnant women led to 96% vaccination coverage among newborns of HBsAg positive mothers in Denmark.** *Vaccine* 2011, 29: 9303-9307.	Prospec-tive cohort study	142,739	Pregnant women	General practitioner clinics	140,376	HBV	98.3	0.3 HBsAg-positive	NA
**Switzer-land**	96. Heininger U et al. **Evaluation of the compliance with recommended procedures in newborns exposed to HBsAg-positive mothers.** *The Pediatric Infectious Disease Journal* 2010, 29: 248-250.	Retro-spective cohort study	27,226	Pregnant women	Hospital maternity units	26,750	HBV	96.6	0.7 HBsAg-positive	NA

Country	Reference	Study design	Sample size (N)	Study population	Study setting	Number of study participants tested	Disease test sought to detect	% tested	Viral hepatitis prevalence (%)	Were study partici-pants informed of test results?
**United Kingdom**	97. Hughes C et al. **Viral screening at the time of each donation in ART patients: is it justified?** *Human Reproduction* 2011, 26: November.	Retro-spective cohort study	Not specified (>12,500)	Women and men utilising assisted reproductive technology	Tertiary hospital referral unit for assisted reproductive technology	HBV: 12,797<br>HCV: 12,762	HBV, HCV	100	0.3 HBsAg-positive<br>0.3 anti-HCV-positive	NA
**Greece**	98. Karatapanis S et al. **Higher rates of chronic hepatitis B infection and low vaccination-induced protection rates among parturients escaping HBsAg prenatal testing in Greece: a 2-year prospective study.** *European Journal of stroenterology and Hepatology* 2012, 24:878–883.	Prospec-tive cohort study	1000	Pregnant women	Hospital obstetric/ gynaecology department	1000	HBV	100	5.3 HBsAg-positive	Yes

Country	Reference	Study design	Sample size (N)	Study population	Study setting	Number of study participants tested	Disease test sought to detect	% tested	Viral hepatitis prevalence (%)	Were study partici-pants informed of test results?
**Germany**	99. Knorr B et al. **Prevalence of hepatitis B virus infection among women at reproductive age at a German university hospital.** *Journal of Clinical Virology* 2008, 42: 422-424.	Cross-sectional study	5518	Pregnant women and women planning to become pregnant	Hospital gynaecology department	5518	HBV	100	1.6 HBsAg-positive	NA
**Turkey**	100. Koruk I et al. **An intervention study to improve HBsAg testing and preventive practices for hepatitis B in an obstetrics hospital.** *TAF Preventive Medicine Bulletin* 2011, 10.	Quasi-experi-mental study	18,709	Pregnant women	Obstetric/ gynaecology hospital	3078	HBV	16.5	0.8 HBsAg-positive	Yes
**Norway**	101. Kristiansen MG et al. **Prevalences of viremic hepatitis C and viremic hepatitis B in pregnant women in Northern Norway.** *Hepato-Gastroenterology* 2009, 56: 1141-1145.	Cross-sectional study	1668	Pregnant women	Ultrasound units	1668	HBV, HCV	100	0.1 HBsAg-positive<br>0.2 HCV-RNA-positive	Yes

Country	Reference	Study design	Sample size (N)	Study population	Study setting	Number of study participants tested	Disease test sought to detect	% tested	Viral hepatitis prevalence (%)	Were study partici-pants informed of test results?
**Ireland**	102. Lambert J et al. **Universal antenatal screening for hepatitis C.** *Irish Medical Journal* 2013, 106: 136-139.	Prospec-tive cohort study	8976	Pregnant women	Hospital	8976	HCV	100	0.9 anti-HCV-positive	NA
**Nether-lands**	103. Op de Coul ELM et al. **Antenatal screening for HIV, hepatitis B and syphilis in the Netherlands is effective.** *BMC Infectious Diseases* 2011; 11.	Retro-spective cohort study	190,141	Pregnant women	NA (national database of screening results from pregnant women)	190,140	HBV	>99.99	0.3 HBsAg-positive	NA
**United Kingdom**	104. Pepas L et al. **Viral screening before each cycle of assisted conception treatment is expensive and unnecessary: a survey of results from UK inner city clinic.** *Human Fertility* 2011, 14:224-229.	Retro-spective cohort study	HBV: 3910<br>HCV: 3953	Women and men utilising assisted reproductive technology	Hospital-assisted reproductive technology unit	HBV: 3910<br>HCV: 3953	HBV, HCV	100	1.7 HBsAg-positive<br>0.4 anti-HCV-positive	NA

Country	Reference	Study design	Sample size (N)	Study population	Study setting	Number of study participants tested	Disease test sought to detect	% tested	Viral hepatitis prevalence (%)	Were study partici-pants informed of test results?
**Portugal**	105. Pereira A et al. **Pre-conception and prenatal care in Braga region.** Journal of Neonatal-Perinatal Medicine Conference: 20th European Workshop on Neonatology Tallinn Estonia. Conference Publication: (var pagings) 2012; 5 (2): 2012.	Retro-spective cohort study	413	Pregnant women	Hospital obstetric department	291	HBV	70.5	NA	NA
**Albania**	82. Resuli B et al. **Epidemiology of hepatitis B virus infection in Albania.** *World Journal of Gastroenterology* 2009, 15:849-852.	Cross-sectional study	640	Pregnant women	NA	640	HBV	100	7.3 HBsAg-positive<br>36.3 anti-HBs-positive	NA
**Germany**	89. Walz A et al. **Vertical transmission of HBV virus (HBV) from mothers negative for HBV surface antigen and positive for antibody to HBV core antigen.** *The Journal of Infectious Diseases* 2009, 200:1227-1231.	Cross-sectional study	2365	Pregnant women who are HBsAg-negative	Maternity clinics	2365	HBV	100	6.2 anti-HBc-positive	Yes

Country	Reference	Study design	Sample size (N)	Study population	Study setting	Number of study participants tested	Disease test sought to detect	% tested	Viral hepatitis prevalence (%)	Were study partici-pants informed of test results?
	People living with HIV									
**Nether-lands**	106. Heiligenberg M et al. **Low prevalence of asymptomatic sexually transmitted infections in HIV-infected heterosexuals visiting an HIV clinic in the Netherlands.** Sexually Transmitted Infections Conference: 19th Biennial Conference of the International Society for Sexually Transmitted Diseases Research Quebec City, QC Canada Conference Start. Conference Publication: (car pagings 2011; 87 (pp A185-A186): July.	Cross-sectional study	245	HIV-positive hetero-sexuals	HIV outpatient clinic	245	HBV, HCV	100	0 HBV-positive (marker not specified)<br>0.4 HCV-positive (marker not specified)	Yes

Country	Reference	Study design	Sample size (N)	Study population	Study setting	Number of study participants tested	Disease test sought to detect	% tested	Viral hepatitis prevalence (%)	Were study partici-pants informed of test results?
**Nether-lands**	107. Matser A et al. **Lifestyle as marker of hepatitis C infection in HIV infected MSM in Amsterdam, the Netherlands.** Sexually Transmitted Infections Conference: 19th Biennial Conference of the Internaitonal Society for Sexually ransmitted Diseases Research Quebec city, QC Canada. Conference Publication: (var pagings 2011; 87 (pp A49): July.	Cross-sectional study	850	HIV-positive MSM	STI outpatient clinic	850	HCV	100	12.7 anti-HCV-positive	NA

Country	Reference	Study design	Sample size (N)	Study population	Study setting	Number of study participants tested	Disease test sought to detect	% tested	Viral hepatitis prevalence (%)	Were study partici-pants informed of test results?
**Bulgaria**	108. Popinova N et al. **Human hepatitis viruses B and C among HIV-infected patients - clinical and epidemiological features.** *Problems of Infectious and Parasitic Diseases*: 37, 2009,1.	Cross-sectional study	48	HIV-positive people	NA	48	HBV, HCV	100	2.1 HBsAg-positive<br>43.8 anti-HCV-positive<br>4.2 HBsAg-positive and anti-HCV-positive (coinfected)	NA
**Germany**	109. Reuter S et al. **Prevalence and characteristics of hepatitis B and C virus infections in treatment-naive HIV-infected patients.** *Medical Microbiology and Immunology* 2011, 200: 39-49.	Cross-sectional study	918	HIV-positive people	NA	918	HBV, HCV	100	4.5 HBsAg-positive<br>10.6 anti-HCV-positive	NA

Country	Reference	Study design	Sample size (N)	Study population	Study setting	Number of study participants tested	Disease test sought to detect	% tested	Viral hepatitis prevalence (%)	Were study partici-pants informed of test results?
**Slovenia**	110. Seme K et al. **Low prevalence of hepatitis B and C infections among HIV-infected individuals in Slovenia: a nationa-wide study, 1986-2008.** *Acta Dermatovenerologica Alpina, Pannonica et Adriatica* 2009, 18: 153-156.	Cross-sectional study	409	HIV-positive people	NA	356	HBV, HCV	87.0	3.9 HBsAg-positive<br>10.7 anti-HCV-positive	NA
**United Kingdom**	111. Steedman NM. **Hepatitis B testing and vaccination in patients recently diagnosed with HIV infection.** *International Journal of STD and AIDS* 2008, 19: 83-84.	Retro-spective cohort study	51	HIV-positive people	Hospital genito-urinary department	31	HBV	60.8	6.5 HBsAg-positive	NA

Country	Reference	Study design	Sample size (N)	Study population	Study setting	Number of study participants tested	Disease test sought to detect	% tested	Viral hepatitis prevalence (%)	Were study partici-pants informed of test results?
**United Kingdom**	112. Turner J et al. **The prevalence of hepatitis C virus (HCV) infection in HIV-positive individuals in the UK - trends in HCV testing and the impact of HCV on HIV treatment outcomes.** *Journal of Viral Hepatitis* 2010, 17: 569-577.	Retro-spective cohort study	31,765	HIV-positive people	NA	20,365	HCV	64.1	8.9 anti-HCV-positive	NA
**Nether-lands**	113. Urbanus AT et al. **Hepatitis C virus infections among HIV-infected men who have sex with men: an expanding epidemic.** *AIDS* 2009, 23: F1-F7.	Cross-sectional study	689	HIV-positive MSM	STI outpatient clinic	689	HCV	100	4.4 anti-HCV-positive	NA
**Switzer-land**	114. Wandeler G et al. **Hepatitis C Virus Infections in the Swiss HIV Cohort Study: a Rapidly Evolving Epidemic.** *Clinical Infectious Diseases* 2012, 55: 1408-1416.	Retro-spective cohort study	11,837	HIV-positive people	NA	11,837	HCV	100	26.4 anti-HCV-positive	NA

Country	Reference	Study design	Sample size (N)	Study population	Study setting	Number of study participants tested	Disease test sought to detect	% tested	Viral hepatitis prevalence (%)	Were study partici-pants informed of test results?
**France**	115. Winnock M et al. **Prevalence of immunity to hepatitis viruses A and B in a large cohort of HIV HCV-coinfected patients, and factors associated with HAV and HBV vaccination.** *Vaccine* 2011, 29: 8656-8660.	Prospec-tive cohort study	1056	People coinfected with HIV and HCV	Hospital wards	1056	HBV	100	2.3 HBsAg-positive	Yes
	General population									
**Turkey**	116. Akcam F et al. **Hepatitis B virus and hepatitis C virus seroprevalence in rural areas of the southwestern region of Turkey.** *Internaitonal Journal of Infectious Diseases* 2009, 13: 274-284.	Cross-sectional study	2852	General population in rural southwest-ern Turkey	NA	2852	HBV, HCV	100	2.5 HBsAg-positive<br>16.2 anti-HBc-positive<br>1.0 anti-HCV-positive	NA

Country	Reference	Study design	Sample size (N)	Study population	Study setting	Number of study participants tested	Disease test sought to detect	% tested	Viral hepatitis prevalence (%)	Were study partici-pants informed of test results?
**United Kingdom**	117. Brant LJ et al. **Diagnosis of acute hepatitis C virus infection and estimated incidence in low- and high-risk English populations.** *Journal of Viral Hepatitis* 2008, 15: 871-877.	Prospec-tive cohort study	3237	General population	NA	3237	HCV	100	0.1 HCV-RNA-positive	NA
**United Kingdom**	118. Brant LJ et al. **Using automated extraction of hepatitis B tests for surveillance: evidence of decreasing incidence of acute hepatitis B in England.** *Journal of Viral Hepatitis* 2012, 140: June.	Cross-sectional study	55,317	General population	NA	55,317	HBV	100	9.4 anti-HBc-positive	NA
**United Kingdom**	119. Brant LJ et al. **Where are people being tested for anti-HCV in England? Results from sentinel laboratory surveillance.** *Journal of Viral Hepatitis* 2008, 15:729-739.	Cross-sectional study	503,060	General population aged 1 and older	NA	503,060	HCV	100	5.5 anti-HCV-positive	NA

Country	Reference	Study design	Sample size (N)	Study population	Study setting	Number of study participants tested	Disease test sought to detect	% tested	Viral hepatitis prevalence (%)	Were study partici-pants informed of test results?
**Italy**	120. Cozzolongo R et al. **Epidemiology of HCV infection in the general population: a survey in a southern Italian town.** *American Journal of Gastroenterology* 2009, 104: 2740-2746.	Cross-sectional study	2195	General population aged 18 and older in a southern Italian town	National Institute of Gastroen-terology and general practitioner practices	2195	HBV, HCV	100	0.5 HBsAg-positive<br>12.0 anti-HBc-positive<br>2.6 anti-HCV-positive	NA
**France**	121. Defossez G et al. **Evaluation of the French national plan to promote screening and early management of viral hepatitis C between 1997 and 2003: a comparative cross-sectional study in Poitou-Charentes region.** *European Journal of Gastroenterology and Hepatology* 2008, 20:367-372.	Cross-sectional study	20,920	General population in a region of southern France	NA	20,920	HCV	100	1.5 HCV-positive (marker not specified)	Yes

Country	Reference	Study design	Sample size (N)	Study population	Study setting	Number of study participants tested	Disease test sought to detect	% tested	Viral hepatitis prevalence (%)	Were study partici-pants informed of test results?
**France**	122. Delarocque-Astagneau E et al. **The impact of the prevention programme of hepatitis C over more than a decade: the French experience.** *Journal of Viral Hepatitis* 2010, 17: 435-443.	Retro-spective cross-sectional study	14,416	General population	Social Security Medical Centres	14,416	HCV	100	NA	Yes
**Italy**	123. Fabris P et al. **Changing epidemiology of HCV and HBV infections in Northern Italy. A survey in the general population.** *Journal of Clinical Gastroenterology* 2008, 42.	Cross-sectional study	965	General population in northeast-ern Italy	NA	965	HBV, HCV	100	1.0 HBsAg-positive<br>2.6 anti-HCV-positive	No<br>(testing was anonymo us)
**Turkey**	124. Ozkan S et al. **Community-based research: cost of the tests used for anti-HBc total seropositivity only and hepatitis B screening.** *Transacions of the Royal Society of Tropical Medicine and Hygiene* 2010, 104: December.	Cross-sectional study	452	General population aged 15 and older in a suburb of Ankara, Turkey	NA	452	HBV	100	42.5 anti-HBc-positive	NA

Country	Reference	Study design	Sample size (N)	Study population	Study setting	Number of study participants tested	Disease test sought to detect	% tested	Viral hepatitis prevalence (%)	Were study partici-pants informed of test results?
**Greece**	125. Zacharakis G et al. **Changes in the epidemiology of hepatitis B virus infection following the implementation of immunisation programmes in northeastern Greece.** *Eurosurveillance* 2009, 14.	Cross-sectional study	6876	General population in northeast-ern Greece	NA	6876	HBV	100	2.0 HBsAg-positive<br>26.1 anti-HBs-positive<br>3.3 anti-HBc-positive	NA

Country	Reference	Study design	Sample size (N)	Study population	Study setting	Number of study participants tested	Disease test sought to detect	% tested	Viral hepatitis prevalence (%)	Were study partici-pants informed of test results?
	Blood donors									
**Turkey**	126. Acar A et al. **The significance of repeat testing in Turkish blood donors screened with HBV, HCV and HIV immunoassays and the importance of S/CO ratios in the interpretation of HCV/HIV screening test results and as a determinant for further confirmatory testing.** *Transfusion Medicine* 2010, 20: June.	Cross-sectional study	72,695	Blood donors	None (blood donor samples evaluated)	72,695	HBV, HCV	100	1.9 HBsAg-positive 0.5 anti-HCV-positive	NA
**Italy**	127. Dettori S et al. **Identification of low HBV-DNA levels by nucleic acid amplification test (NAT) in blood donors.** *Journal of Infection* 2009, 59: 128-133.	Cross-sectional study	22,765	Blood donors	Hospital transfusion centre	22,765	HBV, HCV	100	0.05 HBsAg-positive 0.06 anti-HCV-positive	NA

Country	Reference	Study design	Sample size (N)	Study population	Study setting	Number of study participants tested	Disease test sought to detect	% tested	Viral hepatitis prevalence (%)	Were study partici-pants informed of test results?
**Poland**	54. Ganczak M et al. **Seroprevalence of hepatitis C virus infection among surgical nurses, their patients and blood donation candidates in Poland.** *Journal of Hospital Infection* 2012, 82: 266-270.	Cross-sectional study	801	Blood donors	Blood donation centre	801	HCV	100	0 anti-HCV-positive	Yes
**Turkey**	78. Karaosmanoglu HK et al. **Seroprevalence of hepatitis B: do blood donors represent the general population?** *Journal of Infection in Developing Countries* 2012, 6:181-183.	Retro-spective cohort study	9949	Blood donors	Blood donation centre	9949	HBV	100	1.8 HBsAg-positive	NA
**Poland**	128. Kazmierczak M et al. **Prevalence of viruses HBV, HCV and HIV in blood donors at the Regional Centre of Blood Donation and Blood Treatment in Poznan in the years 2008-2010.** *Annales Universitatis Mariae Curie-Sklodowska, Sectio DDD: Pharmaciqa* 2012, 25: 2012.	Cross-sectional study	148,320	Blood donors	Blood donation centre	148,320	HBV, HCV	100	0.2 HBsAg-positive<br>0.1 anti-HCV-positive	NA

Country	Reference	Study design	Sample size (N)	Study population	Study setting	Number of study participants tested	Disease test sought to detect	% tested	Viral hepatitis prevalence (%)	Were study partici-pants informed of test results?
**Turkey**	129. Oner S et al. **Hepatitis B, hepatitis C, HIV, and VDRL seroprevalence of blood donors in Mersin, Turkey.** *Turkish Journal of Medical Sciences* 2011, 41: 2011.	Retro-spective cohort study	30,716	Blood donors	Blood donation centre	30,716	HBV, HCV	100	2.2 HbsAg-positive<br>0.4 anti-HCV-positive	NA
**Albania**	82. Resuli B et al. **Epidemiology of hepatitis B virus infection in Albania.** *World Journal of Gastroenterology* 2009, 15:849-852.	Cross-sectional study	1664	Blood donors	Blood donation centre	1664	HBV	100	9.1 HBsAg-positive<br>24.3 anti-HBs-positive	NA
**Italy**	130. Romano L et al. **Hepatitis B virus infection among first-time blood donors in Italy: Prevalence and correlates between serological patterns and occult infection.** *Blood transfusion* 2013, 11: 2013.	Prospec-tive cohort study	31,190	Blood donors	Blood transfusion centres	31,190	HBV	100	0.3 HBsAg-positive<br>8.6% anti-HBc-positive	NA

Country	Reference	Study design	Sample size (N)	Study population	Study setting	Number of study participants tested	Disease test sought to detect	% tested	Viral hepatitis prevalence (%)	Were study partici-pants informed of test results?
**Germany**	131. Wiegand J et al. **Autologous blood donor screening indicated a lower prevalence of viral hepatitis in East vs West Germany: epidemiological benefit from established health resources.** *Journal of Viral Hepatitis*, 2009, 16: 743-748.	Cross-sectional study	36,139	Blood donors	Blood transfusion centres	36,139	HBV, HCV	100	0.3 HBsAg-positive<br>0.3 anti-HCV-positive	No

Country	Reference	Study design	Sample size (N)	Study population	Study setting	Number of study participants tested	Disease test sought to detect	% tested	Viral hepatitis prevalence (%)	Were study partici-pants informed of test results?
	Migrants									
**Germany**	132. Heidrich B et al. **High Prevalence of Hepatitis Marker in Immigrant Populations: A Prospective Multicenter Screening Approach in a Real World Setting.** Journal of Hepatology Conference: 47th Annual Meeting of the European Association for the Study of the Liver, International Liver Congress 2012 Barcelona Spain. Conference Publication: (var pagings) 2012; 56(pp S386): April.	Prospec-tive cohort study	1116	Migrants/ immigrants attending primary health care clinics in Germany	Primary care centres	1116	HBV, HCV	100	3.7 HBsAg-positive<br>2.1 anti-HCV-positive	Yes

Country	Reference	Study design	Sample size (N)	Study population	Study setting	Number of study participants tested	Disease test sought to detect	% tested	Viral hepatitis prevalence (%)	Were study partici-pants informed of test results?
**United Kingdom**	133. Jafferbhoy H et al. **The effectiveness of outreach testing for hepatitis C in an immigrant Pakistani population.** *Epidemiology and Infection* 2012, 140: June.	Interven-tion study	250[5]	Immigrant Pakistani community in Scotland (United Kingdom)	Community-based outreach clinics for hepatitis testing	170	HBV, HCV	68.0	0.6 HBsAg-positive<br>4.1 anti-HCV-positive	NA
**United Kingdom**	134. Lewis H et al. **What is the best method of case finding for chronic viral hepatitis in migrant communities?** Gut Conference: British Association for the study of the Liver Annual Meeting, BASL 2011 London United Kingdom Conference publication: (var pagings) 2011; 60 (pp A26): September.	Multi-arm observa-tional study	Arm 1: 5000<br>Arm 2: 1163<br>Arm 3: 600	Pakistan natives living in the United Kingdom	General practitioner/ primary care practices	Arm 1: 0<br>Arm 2: 17<br>Arm 3: 223	HBV, HCV	Arm 1: 0<br>Arm 2: 1.5<br>Arm 3: 37.2	Arm 1: NA<br>Arm 2: 0 HBsAg-positive; 0 anti-HCV positive<br>Arm 3: 1.0 HBsAg-positive; 2.4 anti-HCV positive	NA

[5] Estimated by authors.

Country	Reference	Study design	Sample size (N)	Study population	Study setting	Number of study participants tested	Disease test sought to detect	% tested	Viral hepatitis prevalence (%)	Were study partici-pants informed of test results?
**Greece**	135. Milionis C et al. **Serological markers of hepatitis B and C among juvenile immigrants from Albania settled in Greece.** *European Journal of General Practice* 2010, 16: 236-240.	Cross-sectional study	504	Recent Albanian immigrants aged 10–23 living in Greece	Hospital blood donation department	504	HBV, HCV	100	11.7 HBsAg-positive<br>0.6 anti-HCV-positive	NA
**Nether-lands**	136. Richter C et al. **Hepatitis B prevalence in the Turkish population of Arnhem: Implications for national screening policy?** *Epidemiology and Infection* 2012, 104: April.	Cross-sectional study	709	Turkish migrants in the Netherlands	Mobile laboratory at Turkish community centres and mosques; general practitioner practices	709	HBV, HCV	100	3.0 HBsAg-positive<br>0.4 anti-HCV-positive	Yes

Country	Reference	Study design	Sample size (N)	Study population	Study setting	Number of study participants tested	Disease test sought to detect	% tested	Viral hepatitis prevalence (%)	Were study partici-pants informed of test results?
**Italy**	137. Tramuto F et al. **Serological pattern of hepatitis B, C, and HIV infections among immigrants in Sicily: Epidemiological aspects and implication on public health.** *Journal of Community Health* 2012, 37: 547-553.	Retro-spective cohort study	393	Immigrants attending an outpatient health clinic in Italy	Hospital outpatient clinic for immigrants	393	HBV, HCV	100	10.7 HBsAg-positive<br>5.6 anti-HCV-positive	NA
**Nether-lands**	138. Veldhuijzen IK et al. **Identification and treatment of chronic hepatitis B in Chinese migrants: Results of a project offering on-site testing in Rotterdam, The Netherlands.** *Journal of Hepatology* 2012, 57: December.	Cross-sectional study	1090	Chinese community in the Netherlands	Outreach locations such as Chinese community centres, schools and churches; municipal public health service facilities	1090	HBV	100	0.7 HBsAg-positive<br>20.4 anti-HBc-positive<br>1.1 anti-HCV-positive	Yes

Country	Reference	Study design	Sample size (N)	Study population	Study setting	Number of study participants tested	Disease test sought to detect	% tested	Viral hepatitis prevalence (%)	Were study partici-pants informed of test results?
**Nether-lands**	139. Veldhuijzen IK et al. **Viral hepatitis in a multi-ethnic neighborhood in the Netherlands: results of a community-based study in a low prevalence country.** *International Journal of Infectious Diseases* 2009, 13:e9-e13.	Cross-sectional study	288	Residents of a multi-ethnic neighbourhood in the Netherlands	Community centre in multi-ethnic neighbourhood	HBV: 284<br>HCV: 271	HBV, HCV	HBV: 98.6<br>HCV: 94.1	20.0 anti-HBc-positive<br>1.1 anti-HCV-positive	NA
**Greece**	125. Zacharakis G et al. **Changes in the epidemiology of hepatitis B virus infection following the implementation of immunisation programmes in northeastern Greece.** *Eurosurveillance* 2009, 14.	Cross-sectional study	3746	Selected groups of immigrants in northeast-ern Greece	None<br>(community-based sero-survey conducted by mobile survey unit)	3746	HBV	100	4.4 HBsAg-positive<br>37.8 anti-HBs-positive<br>3.4 anti-HBc-positive	NA

Country	Reference	Study design	Sample size (N)	Study population	Study setting	Number of study participants tested	Disease test sought to detect	% tested	Viral hepatitis prevalence (%)	Were study partici-pants informed of test results?
	Current/former prison inmates									
**Portugal**	140. Barros H et al. **A survey of HIV and HCV among female prison inmates in Portugal.** *Central European Journal of Public Health* 2008, 16: 116-120.	Cross-sectional study	502	Female prison inmates	Prison	415	HCV	88.6	10.6 anti-HCV-positive	Yes
**United Kingdom**	141. Kirwan P et al. **Hepatitis C and B testing in English prisons is low but increasing.** *Journal of Public Health* (Oxf) 2011, 33: 197-204.	Prospec-tive cohort study	318,550[6]	Male and female prison inmates	Prisons	HBV: 8416<br>HCV: 9956	HBV, HCV	HBV: 2.6<br>HCV: 3.1	1.3 HBsAg-positive<br>13.9 anti-HBc-positive<br>24.2 anti-HCV-positive	NA

[6] Estimated by study authors.

Country	Reference	Study design	Sample size (N)	Study population	Study setting	Number of study participants tested	Disease test sought to detect	% tested	Viral hepatitis prevalence (%)	Were study partici-pants informed of test results?
**Portugal**	142. Marques NM et al. **Seroepidemiological survey of transmissible infectious diseases in a Portuguese prison establishment.** *Brazilian Journal of Infectious Diseases* 2011, 15: 272-275.	Cross-sectional study	151	Male prison inmates	Prison	151	HBV, HCV	100	0.7 HBsAg-positive<br>34.4 anti-HCV-positive	Yes
**United Kingdom**	143. Murray E, Jones D. **Audit into blood-borne virus services in Her Majesty's Prison Service.** *International Journal of STD and AIDS* 2008, 19: 347-348.	Retro-spective cohort study	179	Female prison inmates	Prisons	34	HCV	19.0	35.3 anti-HCV-positive	Yes
**Italy**	144. Pontali E, Ferrari F. **Prevalence of hepatitis B virus and/or hepatitis C virus co-infection in prisoners infected with the Human Immunodeficiency Virus.** *International Journal of Prisoner Health* 2008, 4: 77-82.	Prospec-tive cohort study	173	HIV-positive male prison inmates	Prison	164	HBV, HCV	94.8	6.7 HBsAg-positive<br>77.4 anti-HCV-positive	Yes

Country	Reference	Study design	Sample size (N)	Study population	Study setting	Number of study participants tested	Disease test sought to detect	% tested	Viral hepatitis prevalence (%)	Were study partici-pants informed of test results?
**Bulgaria**	145. Popov G et al. **Prevalence of Viral Hepatitis, Human Immunideficiency Virus and Syphilis among Inmates of Bulgarian Prisons.** *Journal of Hepatology* 2013, 58: S229-S407.	Cross-sectional study	658	Male and female prison inmates	Prisons	658	HBV, HCV	100	60.2 anti-HBc-positive<br>28.6 anti-HCV-positive	No<br>(testing was anonymous)

Country	Reference	Study design	Sample size (N)	Study population	Study setting	Number of study participants tested	Disease test sought to detect	% tested	Viral hepatitis prevalence (%)	Were study partici-pants informed of test results?
**Hungary**	86. Treso B et al. **Prevalence and Correlates of HCV, HVB, and HIV infection among prison inmates and staff, Hungary.** *Journal of Urban Health* 2012, 89: 108-116.	Cross-sectional study	4894	Male and female prison inmates	Prison	4894	HBV, HCV	100	1.5 HBsAg-positive<br>4.9 anti-HCV-positive	NA
**France**	146. Verneuil L et al. **Prevalence and risk factors of the whole spectrum of sexually transmitted diseases in male incoming prisoners in France.** *European Journal of Clinical Microbiology and Infectious Diseases* 2009, 28: 409-413.	Cross-sectional study	597	Male prison inmates	Prisons	442[7]	HBV, HCV	74[8]	0.3 HBV-positive (marker not specified)<br>4.8 anti-HCV-positive	NA

[7] Approximation.
[8] Approximation

Country	Reference	Study design	Sample size (N)	Study population	Study setting	Number of study participants tested	Disease test sought to detect	% tested	Viral hepatitis prevalence (%)	Were study partici-pants informed of test results?
	Health care workers									
**Poland**	52. Flisiak R et al. **Prevalence and risk factors of HCV infection in Poland.** *European Journal of Gastroenterology and Hepatology* 2011, 23: 1213-1217.	Cross-sectional study	9029	Health care workers	Hospitals and outpatient clinics	9029	HCV	100	1.4 anti-HCV-positive<br>0.4 HCV-RNA-positive	NA
**Poland**	54. Ganczak M et al. **Seroprevalence of hepatitis C virus infection among surgical nurses, their patients and blood donation candidates in Poland.** *Journal of Hospital Infection* 2012, 82: 266-270.	Cross-sectional study	414	Surgical nurses and midwives	Hospitals	414	HCV	100	1.4 anti-HCV-positive	Yes
**Germany**	64. Ross RS et al. **Transmission of hepatitis C virus in an orthopedic hospital ward.** *Journal of Medical Virology* 2009, 81: 249-257.	Retro-spective cohort study	104	Health care workers in a hospital orthopaedic ward	Hospital orthopaedic ward	104	HCV	100	1.0 HCV-positive (marker unreported)	Yes

Country	Reference	Study design	Sample size (N)	Study population	Study setting	Number of study participants tested	Disease test sought to detect	% tested	Viral hepatitis prevalence (%)	Were study partici-pants informed of test results?
**Poland**	147. Slusarczyk J et al. **Cross-sectional, anonymous screening for asymptomatic HCV infection, immunity to HBV, and occult HBV infection among health care workers in Warsaw, Poland.** *Przeglad Epidemiologiczny* 2012, 66:445-451.	Cross-sectional study	961	Health care workers	Hospitals	961	HBV, HCV	100	15.7 anti-HBc-positive<br>1.7 anti-HCV-positive<br>0.3 HCV-RNA-positive	No<br>(testing was anony-mous)
**Greece**	148. Topka D et al. **Prevalence of hepatitis B in haemodialysis nursing staff in Athens.** *Journal of Renal Care* 2012, 38: 76-81.	Cross-sectional study	216	Haemodialy-sis nurses	Haemodialy-sis units	216	HBV	100	0.5 HBsAg-positive	NA
**Nether-lands**	149. Zaaijer HL et al. **Hepatitis C virus infection among transmission-prone medical personnel.** *European Journal of Clinical Microbiology and Infectious Diseases* 2012, 31: 1473-1477.	Cross-sectional study	729	Health care workers	Academic medical centre	729	HCV	100	1.4 anti-HCV-positive	No<br>(testing was anony-mous)

Country	Reference	Study design	Sample size (N)	Study population	Study setting	Number of study participants tested	Disease test sought to detect	% tested	Viral hepatitis prevalence (%)	Were study partici-pants informed of test results?
	Men who have sex with men									
**Croatia**	150. Bozicevic I et al. **Prevalence of HIV and sexually transmitted infections and patterns of recent HIV testing among men who have sex with men in Zagreb, Croatia.** *AIDS Behavior* 2009, 13: 303-309.	Cross-sectional study	387	MSM	Hospital-based HIV centre	387	HBV, HCV	100	0.8 HBsAg-positive<br>44.0 anti-HBs-positive<br>6.9 anti-HBc-positive	NA
**Italy**	151. Di Benedetto MA et al. **Prevalence of sexually transmitted infections and enteric protozoa among homosexual men in western Sicily (south Italy).** *Journal of preventive Medicine and Hygiene* 2012, 53: December.	Cross-sectional study	74	MSM	NA	74	HCV	100	0 HCV-positive (marker not specified)	Yes

Country	Reference	Study design	Sample size (N)	Study population	Study setting	Number of study participants tested	Disease test sought to detect	% tested	Viral hepatitis prevalence (%)	Were study partici-pants informed of test results?
**Belgium**	152. Platteau T et al. **Voluntary outreach counselling and testing for HIV and STI among men who have sex with men in Antwerp.** *Acta Clinica Belgica* 2012, 67: 172-176.	Cross-sectional study	137	MSM	Gay sauna; gay fetish club	137	HBV, HCV	100	0.7 HCV-positive (marker not specified)	Yes
**United Kingdom**	153. Scott C et al. **Unselected hepatitis C screening of men who have sex with men attending sexual health clinics.** *Journal of infection* 2010; 60/5):351-353	Cross-sectional study	5230	MSM	Sexual health clinics	3365	HCV	68.6	0.7 anti-HCV-positive	Yes
**Nether-lands**	44. van Houdt R et al. **Unexpectedly high proportion of drug users and men having sex with men who develop chronic hepatitis B infection.** *Journal of Hepatology* 2012, 57:529-533.	Retro-spective cohort study	1862	MSM	Public Health Service Amsterdam	1862	HBV	100	12.0 HBsAg-positive<br>44.0 anti-HBc-positive<br>1.2 anti-HCV-positive	No

Country	Reference	Study design	Sample size (N)	Study population	Study setting	Number of study participants tested	Disease test sought to detect	% tested	Viral hepatitis prevalence (%)	Were study partici-pants informed of test results?
Children born to HBsAg+ mothers										
**Nether-lands**	154. Boot HJ et al. **Persistent and transient hepatitis B virus (HBV) infections in children born to HBV-infected mothers despite active and passive vaccination.** *Journal of Viral Hepatitis* 2010, 17:872-878.	Cross-sectional study	2280	Infants born to HBsAg-positive mothers	NA	1743	HBV	75.4	0.7 HBsAg-positive	NA
**Italy**	155. Bracciale L et al. **Impact of hepatitis B vaccination in children born to HBsAg-positive mothers: a 20-year retrospective study.** *Infection* 2009, 37: 340-343.	Retro-spective cohort study	100	People born to HBsAg-positive mothers who received post-exposure prophylaxis, 1984–2004	NA	100	HBV	100	2.0 anti-HBc-positive	NA

Country	Reference	Study design	Sample size (N)	Study population	Study setting	Number of study participants tested	Disease test sought to detect	% tested	Viral hepatitis prevalence (%)	Were study partici-pants informed of test results?
**Nether-lands**	156. Hahne S et al. **Prevention of perinatal hepatitis B virus transmission in the Netherlands, 2003–2007: Children of Chinese mothers are at increased risk of breakthrough infection.** *Vaccine* 2012, 30: 21.	Cross-sectional study	2657	Infants born to HBsAg-positive mothers	NA	2121	HBV	79.8	0.6 HBsAg-positive	NA
